# 

**Published:** 1894-07

**Authors:** 


					﻿CONTENTS.
ORIGINAL ARTICLES—
Removal of Septic Infectious Matter with a View of Relieving Phleg-
masia Alba Polens. By Geo. H. Noble, M.D., Atlanta, Ga.............. 349
The Marriage of Syphilitics. By William G. Porter, M.D., Philadel-
phia, Pfu.........................................................  -354
Rare Cases in Obsterics. By William F. Holt, M.D, Macon, Ga......... 359
Early Operation in Strangulated Hernia. By VVilliam Perrin Nicolson,
M.D.,,Atlanta, Ga................................................. 362
The Treatment of Inguinal Hernia in Children. By Samuel E. Mil-
iken, M.D., New York................................................  365	.
SOCIETY REPORTS—
Clinical Society of Maryland....:................................... 367
Gynecological and Obstetrical Society of Baltimore.................. 369
BOOK REVIEWS—
Transactions of the Southern Surgical and Gynecological Association,
Vol. VT. Sixth Session, held at New Orleans, La. Published by the
Association........................ ......................................................... 380
A Text-book of the Theory and Practice of Medicine. By American
Teachers................................................................................,. 381
SELECTIONS AND ABSTRACTS—
The Causes and Treatment of Infantile Diarrhea...................... 382
Catarrhal Urethritis in Boys.......%.............................................. 384
Strangulated Hernia................................................  385
Relief of Ingrowing Toe-Nail..:..................................... 386
SELECTIONS AND ABSTRACTS—Continued—
The Treatment of Hysterical Aphonia............................  387
A Case of Concealed Accidental Hemorrhage during the First Stage
, of Labor, with Recovery of the Mother under Conservative Treat-
ment ................................................'......   387
Treatment of Typh'oid Fever by Carbolic Acid and Chloroform...... 388
Death in the Tooth-brush...... ...*........?................ ,.. 389
A Rational Treatment of Prostatic Obstruction in Old Men....:....	389
Experience with Ether Narcosis up to the Present Time. ......... 390
Hermaphroditism.....'.'.... ................................     391
The Erect Posture for Gynecological Examination.....'.........   391
A Slight Mistake................„.....'.'.i'.,...	....?..'...... 392
Prevention of Iodism.....-...............  '...............      392
A Mechanical Device for Illustrating the Movement of the Lung in
Penetrating Wounds of the Chest.. .«.’...... .....;........... 398
What is a Blush....,..'...............■........................  398
Ivy Poisoning  ............. /.......'...........,<.,•,,1....... 393
EDITORIAL.........1........  J....................... ,	J........... 394
EDITORIAL NOTES..................................................... 398
PRESCRIPTION DEPARTMENT...........................'........:.....398,	399
Myalgia—Fermentative Dyspepsia—Cracked Nipples—Application for
Chronic Ulcer—Inflammatory Earache—Chronic Cystitis - Vomiting
of Pregnancy—Painful Dentition—A Carbonated Laxative—Typhoid
Fever.
SPECIAL NOTES........ .................*..........,.400,	402, 403, 405, 408
				

